# Validation and Investigation on the Mechanical Behavior of Concrete Using a Novel 3D Mesoscale Method

**DOI:** 10.3390/ma12162647

**Published:** 2019-08-20

**Authors:** Yuhang Zhang, Zhiyong Wang, Jie Zhang, Fenghua Zhou, Zhihua Wang, Zhiqiang Li

**Affiliations:** 1 Institute of Applied Mechanics, Taiyuan University of Technology, Taiyuan 030024, China; 2 Shanxi Key Lab. of Material Strength & structural Impact, College of Mechanical and Vehicle Engineering, Taiyuan 030024, China; 3 MOE Key Laboratory of Impact and Safety Engineering, Ningbo University, Ningbo 315211, China

**Keywords:** mesoscale model of concrete, Voronoi tessellation, damage plasticity model, Interfacial Transitional Zone (ITZ) phase

## Abstract

The mechanical performance of concrete is strongly influenced by the geometry and properties of its components (namely aggregate, mortar, and Interfacial Transitional Zone (ITZ)) from the mesoscale viewpoint, and analyzing the material at that level should be a powerful tool for understanding macroscopic behavior. In this paper, a simple and highly efficient method is proposed for constructing realistic mesostructures of concrete. A shrinking process based on 3D Voronoi tessellation was employed to generate aggregates with random polyhedron and grading size, and reversely, an extending procedure was applied for ITZ generation. 3D mesoscale numerical simulation was conducted under a quasi-static load using an implicit solver which demonstrated the good robustness and feasibility of the presented model. The simulated results resembled favorably the corresponding experiments both in stress–strain curves and failure modes. Damage evolution analysis showed that the ITZ phase has profound influence on the damage behavior of concrete as damage initially develops from here and propagates to mortar. In addition, it was found that tensile damage is the principal factor of mortar failure while compressive damage is the principal factor of ITZ failure under compression.

## 1. Introduction

The composite behavior of concrete is very complex due to the fact that its overall properties are controlled by the characteristics of different components. Up to now, many details such as strain softening, micro-crack propagation, and failure mechanisms are still far from being fully understood. As a highly nonhomogeneous artificial composite material, concrete generally consists of two raw materials, namely, coarse aggregates and mortar matrix with dissolved fine aggregates, and the derivative component, namely, the Interfacial Transition Zone (ITZ) between coarse aggregates and the mortar matrix. A mesoscale model permits a direct description of the material heterogeneity, therefore, analyzing the material at mesoscale level should be a powerful tool for understanding and predicting the observed macroscopic behavior.

A mesoscale model of concrete can be established in different numerical ways. One approach is to simulate the shape, size, and distribution of the coarse aggregates directly in the 2D and 3D domains. A number of studies on the 2D method of mesoscale modeling can be found in previous reports [1,2,3,4,5,6,7,8,9,10,11]. Though more realistic aggregate shapes such as the random polygon have been achieved in 2D domain, 2D mesoscale models are not accurate to some extent and have an inherent limitation in representing realistic stress and strain conditions [12,13]. Then the numerical 3D model was developed. Bargmann et al. [14] reported a lot of techniques of art Representative Volume Element (RVE) generation for heterogeneous materials, which is comprehensive and valuable for establishing a mesoscale concrete model. Random dispersion of spherical regions was used by Segurado et al. [15] to investigate the effect of particle clustering on the mechanical properties of composites. Eckardt et al. [16] and Yu et al. [17] presented a new algorithm for the mesoscale model with random distribution of heterogeneous particles. Nevertheless, the spherical or ellipsoidal particles do not agree well with the real particle shape in the actual materials. Then polyhedral particles [9,18] were used to establish the mesoscale model of concrete. Wang et al. [19] developed “taking-and-placing” method to generate 3D mesoscale structures with randomly distributed polyhedral aggregates. Interaction and overlap checking algorithms for distributed aggregates and voids were also proposed. Zhang et al. [20] proposed the random walking algorithm for establishing physically feasible structures of concrete in the three-dimensional domain. This approach is able to simulate the movements of aggregates by both translational and rotational actions. Consequently, higher degrees of aggregate content can be well achieved. Yet, judgment between aggregates for avoiding interactive overlap needs a complex algorithm in programming and the time to be spent is much longer especially for a higher volume content of particles. Voronoi tessellations, and their variants, provide a more simple method able to reproduce the non-regularity polyhedron [21,22]. In early 1992, Stankowski [23] first considered subdividing the Voronoi polygons as aggregates in 2D space. Aggregate is generated by shrinking the size of polygons in a random manner and at the same time controlling the aggregate area fraction. Caballero et al. [24] generated a particle array using Voronoi tessellation. To obtain discrete aggregate, the geometry polyhedrals were shrunk and the spaces between aggregates were meshed with finite elements representing mortar phase. Galindo-Torres et al. [25] introduced a new way to model particles using Voronoi-spheroid polyhedral tessellation. Then, a natural and consistent method was developed to simulate bonding between granular particles. It can be found that Voronoi techniques have been widely accepted for the generation of aggregates. However, there are some inherent defects for traditional Voronoi models, for example, the grading of aggregates is hard to achieve and the distance between cells cannot be controlled easily. The other approach is to reconstruct particles based on scanning technology [26]. Man and Mier [27] obtained the concrete structure containing varying amounts of aggregates from CT-scans to analyze the size effect. Liu et al. [28] reconstructed a microstructural model of asphalt mixture based on X-ray CT scans and the relationship between aggregate angularity and mechanical responses of asphalt mixture was investigated. The main limitation of the technique is that it is very time-consuming and expensive to obtain meaningful analyses by a series procedure such as preparing, fabricating, cutting, and scanning.

In these studies, the researchers mainly focus on coarse aggregate phase generation, however, the establishment of the ITZ phase, which is also an important component in mesoscale models, has not really been mentioned above.

It is well known that the ITZ plays an important role in the macro properties of concrete. In this zone, the cement particles are unable to bind intimately with the relatively large particles of the aggregate; consequently, the ITZ has much higher porosity than that of the hardened cement phase farthest from the aggregate particles [29]. As a result, the ITZ phase is considered the weakest link in concrete and its influence on the overall behavior of concrete still needs further research. Many researchers [19,24,30,31,32] have proposed zero thickness cohesive interfaces between aggregate and mortar as the replacement of ITZ via an in-house program. Because of its simple formation, the cohesive zone model has wide applications in the fracture of concrete, especially in crack propagation and the quasi-brittle process. In realistic concrete, the ITZ phase can be observed and plays an important role in the macro properties of concrete though the scale of ITZ is much less than the aggregate size. On the one hand, solid elements applied in ITZ can better reflect the practical ITZ shape than a zero thickness cohesive zone. On the other hand, damage evolution in the ITZ phase can be clearly observed under both compressive and tensile stress, which could well illustrate the mechanism of concrete failure [33,34]. It is inevitable to generate more realistic three-dimensional meshes for the ITZ to get better numerical results [35,36]. Meshing the ITZ in tetrahedron would generate large elements [37] as the ITZ size is much smaller than coarse aggregates. This would be super time-consuming and cannot be accepted in meso-level models of concrete. Bernard et al. [38] mapped the 3D mesoscale model into finite element and the thickness of the ITZ was the minimum voxel length. The mapping element does have a lot of advantages such as more robustness and being less time-consuming, but the true shape of concrete is still not reflected. Li [35] proposed an aggregate-expansion method to mesh the ITZ in brick elements with a limited number of elements and performed it on a 3D concrete specimen from CT images. Although the ITZ phase could be meshed in the acceptable way, the computational adaptability for different material models should be further improved.

Finite element (FE) computation is another problem for mesoscale modeling due to the complex mixture of three different materials. For quasi-static loading conditions, dynamic transient analysis was employed in most papers [18,19,33,36]. It determines a solution to the dynamic equilibrium equation by kinematic state from the previous increment; as a result, inertia and spurious oscillation in the simulation could lead to uncontrollable deviations, especially in damage analysis.

In this study, a simple and less time-consuming method was developed to generate the three-phase mesoscale model of concrete based on a 3D Voronoi tessellation. A shrinking process was proposed for generating independent aggregates with random shape and grading size. An “EDGEdeletion” program was conducted to resolve some problems in geometrical feature. The opposite approach called extending process was presented to generate the ITZ in a certain thickness. The geometrical model can be effectively incorporated within the simulated process using implicit solver. The mesoscale model was verified against standard experimental observations under quasi-static compression and tension. Furthermore, the model was then applied to investigate the initiation and evolution of damage especially for the ITZ phase.

## 2. Generation of Mesostructured Model

### 2.1. Geometry of Coarse Aggregate

The convex polyhedral geometry is generated based on the Voronoi technique. A specific region, whose space is larger than the required specimen size, is divided into seamlessly connected cells with Voronoi tessellation as N seeds (nuclei) placed in advance randomly within the region. $S_{i}$ denotes the seed, and its corresponding Voronoi cell $V_{i}$ consists of every point P (P∈D) in the region whose distance to $S_{i}$ is less than or equal to its distance to any other seed [39]:

(1) $$V_{i}=\left\{P\in D\;|\;d\left(P,S_{i}\right)\leq \;d\left(P,S_{j}\right)\;,\;i\neq j\right\}$$

where d is the Euclidean distance, which can be expressed as

(2) $$d\left(P,S\right)=\sqrt{{\left(P_{x}-S_{x}\right)}^{2}+{\left(P_{y}-S_{y}\right)}^{2}+{\left(P_{z}-S_{z}\right)}^{2}}$$

In order to avoid sharp corners, it is necessary to control the minimum distance between any two of the nuclei according to the following principle:

(3) $$\delta_{\min}=\left(1-K\right)\delta_{0}$$

where $\delta_{0}$ is the average distance between two nuclei calculated as $\delta_{0}=\frac{\sqrt{6}}{2}{\left(\frac{V}{\sqrt{2}N}\right)}^{\frac{1}{3}}$, and K is the irregular degree of convex polyhedron, for example, K=1 indicates that the shape of cell is completely random, while K=0 indicates that the cell is a regular polyhedron. In this study, K=0.2 is adopted [40].

**Figure 1:**
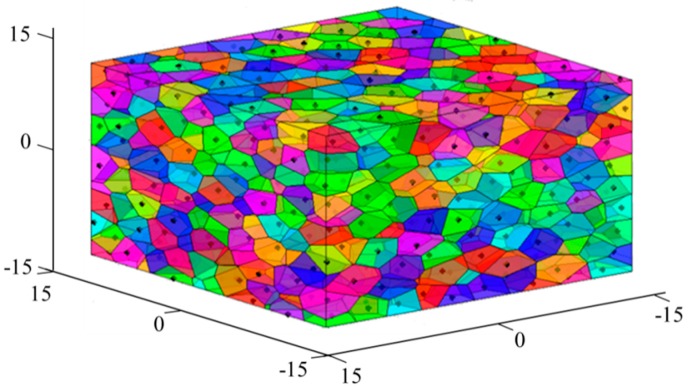
Resulting 3D Voronoi polyhedrons when K = 0.2.

As an initial modeling of aggregates, the 3D Voronoi diagram is established using MATLAB (The MathWorks Inc, St, Natick, MA, USA) and plotted in Figure 1. Each of the cells meets the convexity of a 3D polyhedron and can be considered as an aggregate. In order to separate these connected cells into independent aggregates and leave space for the mortar phase, the shrinking process is used on the random polyhedron. For each cell, the vector $\vec{v}$ from vertex $p_{i}$ to its corresponding seed $S_{i}$ can be given as $\vec{v}=\left[\begin{array}{c} x_{S_{i}}-x_{p_{i}}\\ y_{S_{i}}-y_{p_{i}}\\ z_{S_{i}}-z_{p_{i}} \end{array}\right]$ and shortened by the shrinking factor q (0<q<1). The updated vertex $p_{i}^{\prime }$ is obtained by

(4) $$p_{i}^{\prime }=p_{i}+q\vec{v}$$

**Figure 2:**
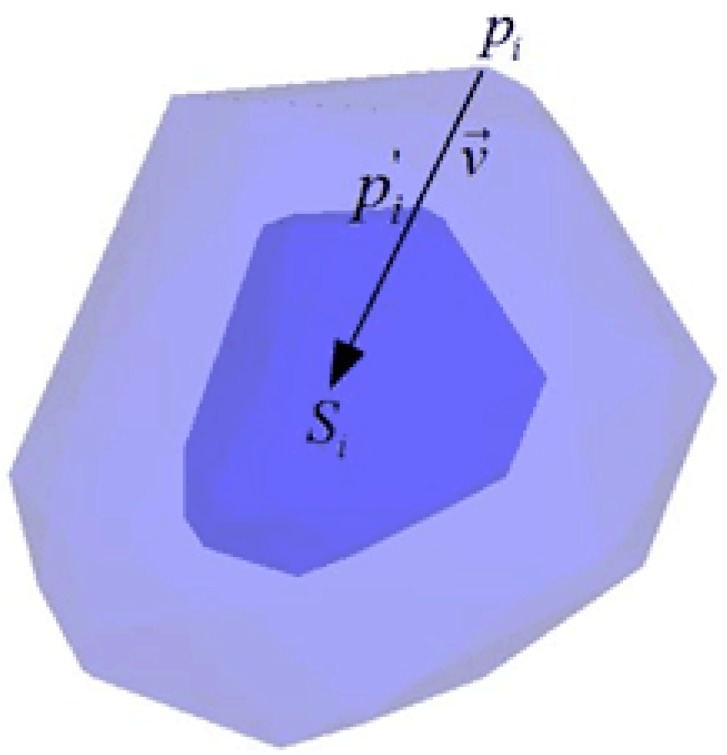
Schematic diagram of shrinkage for a single aggregate.

Figure 2 plots the shrinking scheme of a single cell. It should be mentioned that the shrinking factor should be the same within one aggregate, otherwise, the convexity of the polyhedron will be destroyed. Moreover, a different shrinking factor can be taken for various aggregates to meet the requirements of grading. The Fuller curve, developed by Fuller and Thompson, is most commonly used to describe the optimum size distribution of aggregates, and can be expressed as

(5) $$P\left(d\right)=100{\left(\frac{d}{d_{\max}}\right)}^{n}$$

where P(d) is the accumulated percentage that pass the sieve with aperture diameter d, $d_{\max}$ is the maximum size of aggregates, and n is an exponent in the range of 0.45–0.7. Generally, n is taken as 0.5.

The volume of aggregates within each grading segment $\left[d_{i},d_{i+1}\right]$ can be described as

(6) $$V_{\mathrm{agg}}\left[d_{i},d_{i+1}\right]=\frac{P\left(d_{i+1}\right)-P\left(d_{i}\right)}{P\left(d_{\max}\right)-P\left(d_{\min}\right)}\times P_{\mathrm{agg}}\times V$$

where $d_{\max}$ and $d_{\min}$ are the maximum and minimum sizes of aggregates, respectively; $P_{\mathrm{agg}}$ is the volume fraction of aggregates, and V is the volume of the entire sample.

Based on the volume of each Voronoi polyhedron, the number of each grading segment can be calculated and then the shrinking scale can also be obtained. The shrinking factor q for a different segment $\left[d_{i},d_{i+1}\right]$ is random and can be expressed as

(7) $$q\left[d_{i},\;d_{i+1}\right]=1-\frac{d_{i+1}}{d_{\max}}+\omega \frac{d_{i+1}-d_{i}}{d_{\max}}$$

where ω is the uniform random variable between 0 and 1.

**Figure 3:**
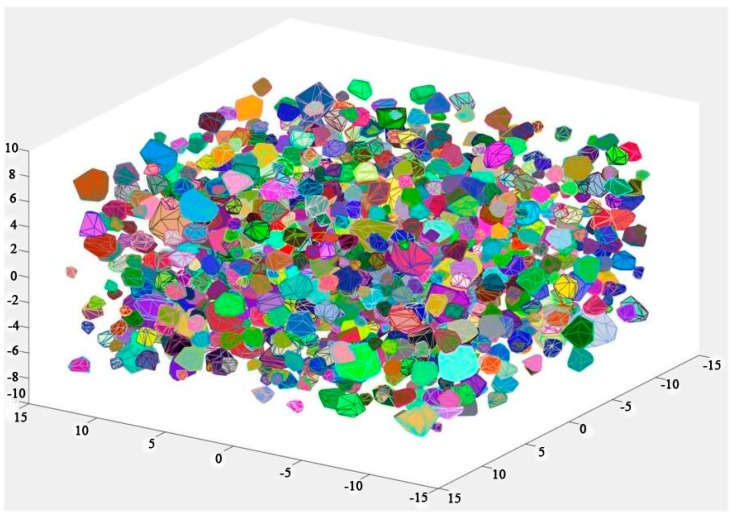
Separated aggregates in a certain space by shrinking process.

**Figure 4:**
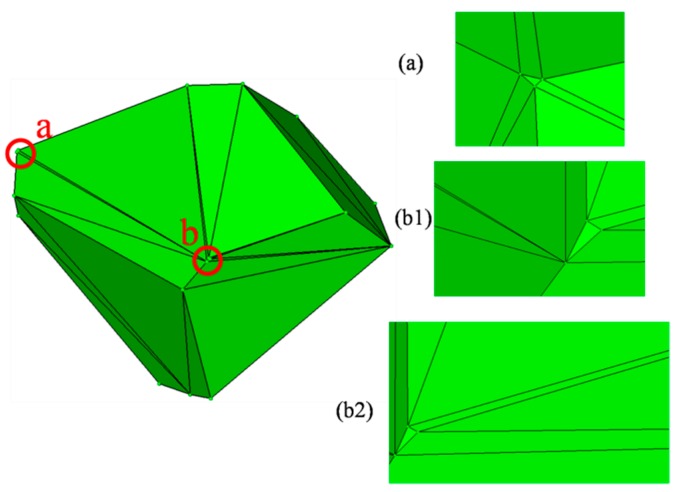
Defects in geometry features after separated process: (**a**) Small area at location “a”, (**b1**) short sides at location “b”, (**b2**) sharp triangle with a small angle and long sides at location “b”.

**Figure 5:**
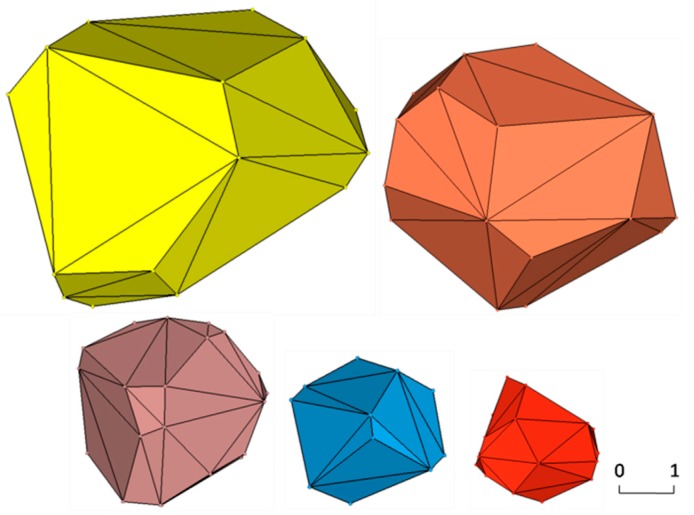
Regeneration aggregate cell with grading size.

The updated vertexes of each cell are connected, complying with the Delaunay rule, and aggregates with random shape and grading size are generated. A result of this process is shown in Figure 3. A graded result of this process is shown in Table 1. The graded aggregates are set as 12.7 mm, 9.5 mm, 4.75 mm, and 2.36 mm according with the classical grading distribution in concrete by Hirsch [41]. Figure 4 marks some details of a single aggregate selected in Figure 3. The convex hull has some near points and small areas with extremely long or short edges, which has a great influence on further processes such as forming the ITZ and meshing. Moreover, a highly irregular ratio would generate an enormous amount of elements, even poor quality elements, in the finite element method and it is the main cause of computing interruption. To deal with this problem, a program called “EDGEdeletion” inserts the main routine developed by MATLAB. The procedure for modifying the geometry feature is described in detail below:

(1) Extract the x/y/z coordinates of all the surface nodes.

(2) Find the points according to the mutual distance that is less than a certain value. It may be that the point in this feature is more than two points like P_1_, P_2_ and P_3_....

(3) Replace the coordinates including x, y, and z to the same point found in Step 2 above like P_1_ = P_2_, P_1_ = P_3_…, then delete the points with only one point reserved, for example, reserving the point P_1_ and removing the points P_2_ and P_3_.

(4) Regenerate the new closed hulls and check whether the distance between points is more than the certain value; if not, recycle the procedure from Step 1 until the answer is yes. Figure 5 depicts the aggregates with grading size after conducting the “EDGEdeletion “program. The scale bar gives a measuring standard for particles after the procedure of shrinkage and EDGE deletion, and aggregates with different sizes can be vividly plotted in the figure.

### 2.2. Generation of ITZ

The geometry of the ITZ is generated by an extending process, which is an opposite way to the form of aggregate. As an updated aggregate vertex $p_{i}^{\prime }$ is computed according to Equation (4), the new extending factor η (0<η<1) is defined and the ITZ vertex $p_{i}^{\prime \prime }$ is obtained by

(8) $$p_{i}^{\prime \prime }=p_{i}^{\prime }-\eta \vec{v}$$

**Figure 6:**
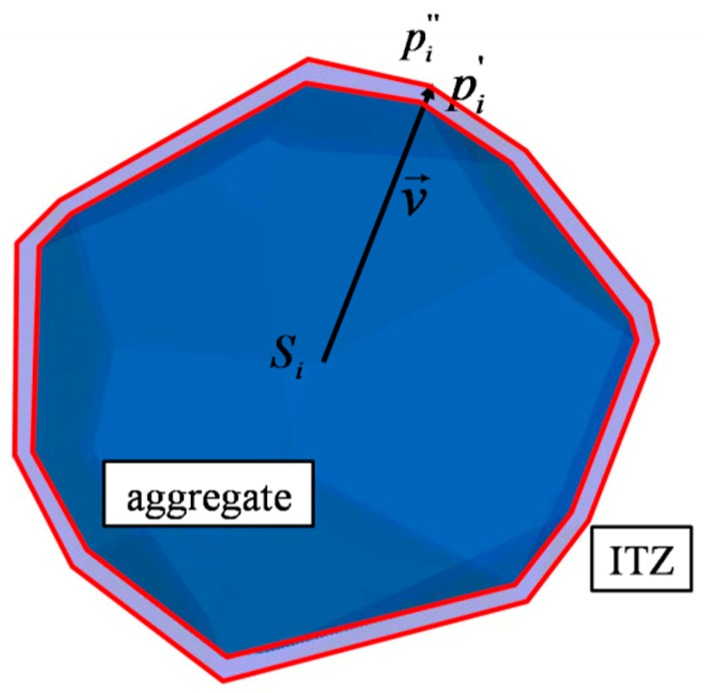
Schematic diagram of generating the Interfacial Transitional Zone (ITZ).

**Figure 7:**
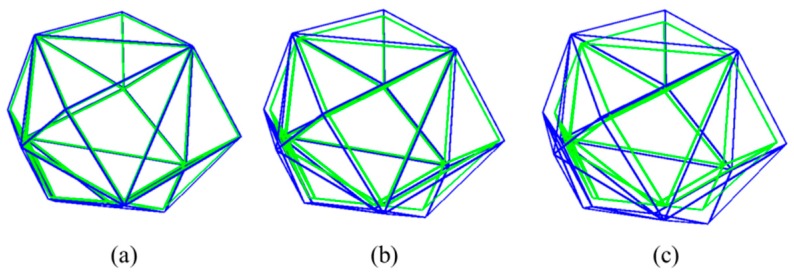
Geometry outlines of different ITZ layers: (**a**) 0.1 mm thickness η=0.05, (**b**) 0.25 mm thickness η=0.125, (**c**) 0.5 mm thickness η=0.25.

Figure 6 shows the schematic diagram for forming the ITZ. The extending method could be another less time consuming way to generate the ITZ phase, which is different from the aggregate-expansion method [35] and the equivalent solid approach [36]. In traditional methods, time consumption is large because the detection criterion is necessary to judge the overlap for aggregates one by one. This process generates a certain thickness of ITZ at the same time without intersection judgment. Zhang et al. [42] gives a comparison of different algorithms and the result shows that the present method has an obvious advantage in saving time. Also, different thickness of ITZ can be generated when the extending factor η  is changed (Figure 7). The green lines are the out contour of the solid aggregate, and the blue lines are the out contour of the solid ITZ phase.

## 3. 3D Mesoscale Finite Element Discretization

**Figure 8:**
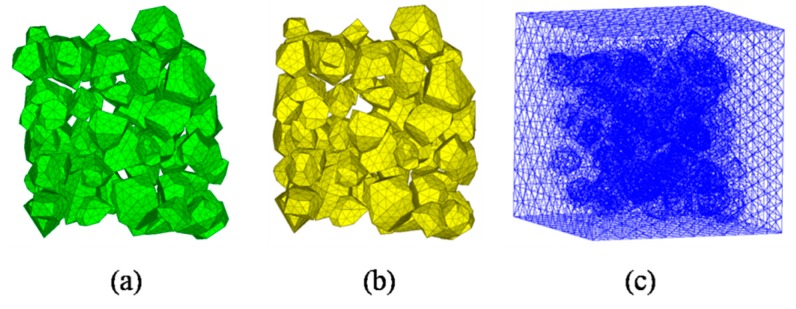
Three-phase mesoscale concrete FE model: (**a**) Aggregate phase, (**b**) ITZ phase, (**c**) mortar phase.

The characteristic size of the ITZ is quite small compared with the coarse aggregate used in the laboratory. It is unacceptable to mesh the ITZ using tetrahedral elements because millions of elements would be generated and the computational efficiency of the model will drop dramatically. The wedge element with integration 15-nodes was adopted to decrease the numbers of ITZ elements and ensure the computing accuracy, while the tetrahedral elements were adapted to aggregates and mortar phase. Figure 8 plots the meshing elements of a three-phase concrete model, respectively.

**Figure 9:**
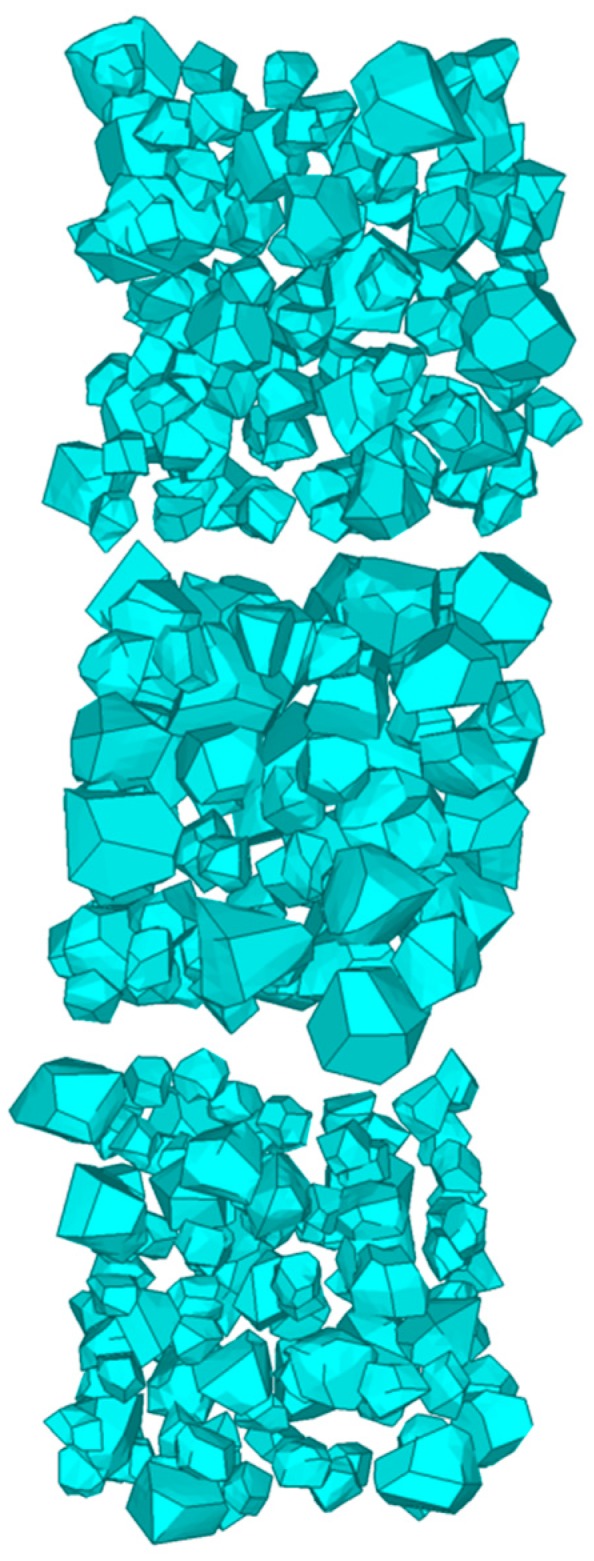
A generated model before free falling.

**Figure 10:**
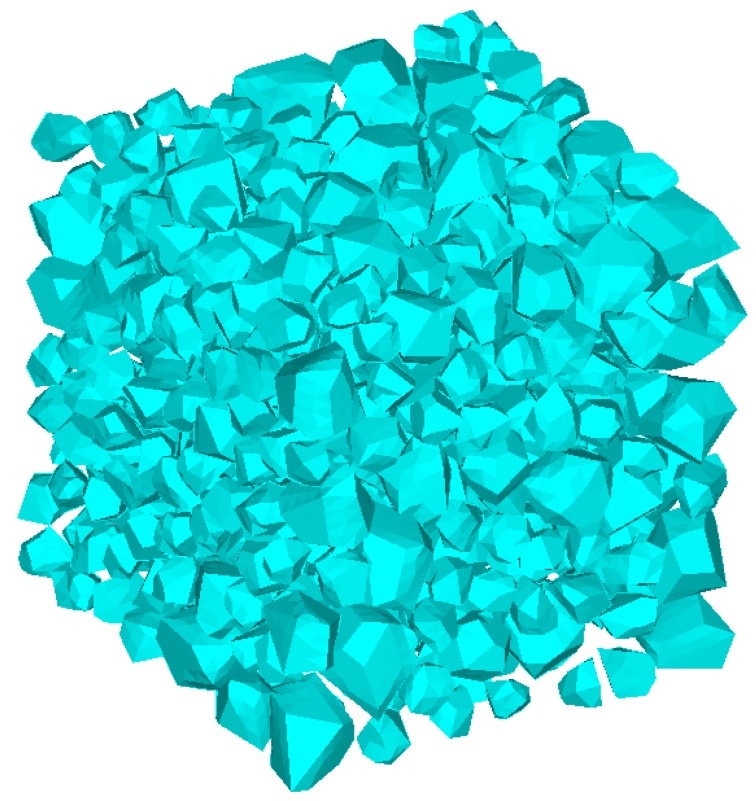
A simulated model after free falling.

A common challenge to most existing mesostructure models of concrete is low aggregate packing density. In order to improve the aggregate volume ratio, the free falling of randomly distributed particles in finite element method is applied [43]. The “free falling” procedure is conducted after mesh generation. The falling process is carried out by using FE code LS-dyna. Free fall acceleration is applied to the particles. Every single grain is regarded as a single part (Figure 9) and then falls together. Figure 10 shows the simulated result after this process with a particle volume fraction of 40%, while the corresponding value is 10%. Additionally, the falling process in this study contains meshed ITZ rather than only aggregate phase.

**Figure 11:**
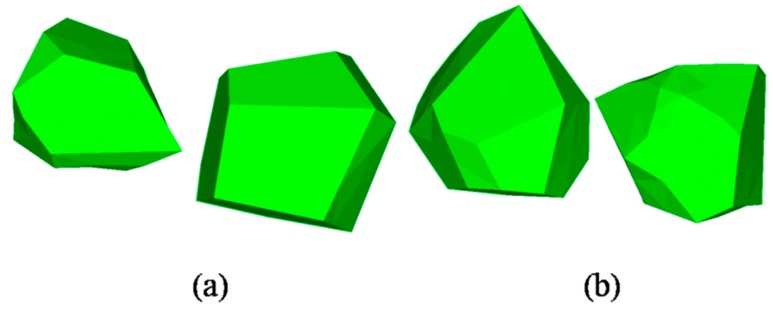
Different relative position of adjacent grains: (**a**) a corner on the surface (**b**) a corner on another corner.

The surfaces of an aggregate produced by the shrinkage procedure may have a face-to-face feature with the adjacent surfaces of other aggregates. These drawbacks will be overcome after the free falling process. The condition, like a corner of one grain pointing on the surface of another one, showed in Figure 11, is common in realistic concrete specimens.

**Figure 12:**
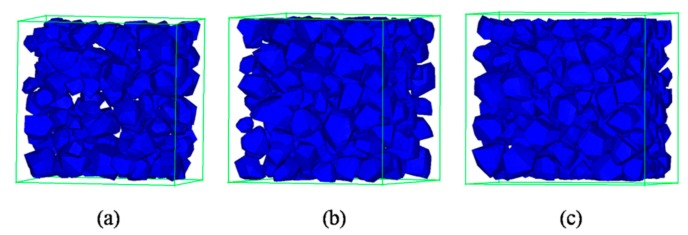
Different aggregate content for cubic specimen: (**a**) 24.96%, (**b**) 37.38%, (**c**) 44.45%.

Figure 12 gives different volume ratios of graded aggregates after the falling process. The different volume ratios of aggregate can be accomplished by changing the times of the “free falling” procedure. Higher volume ratios will be achieved after a repeated falling process. The diagrams illustrate that the falling procedure not only increases the aggregate content, but also perfects the distribution of grain phase. The “perfect” means that the distribution of aggregate becomes more random and compact. The space between each grain is similar, not too big or too small, which is closer to realistic concrete.

**Figure 13:**
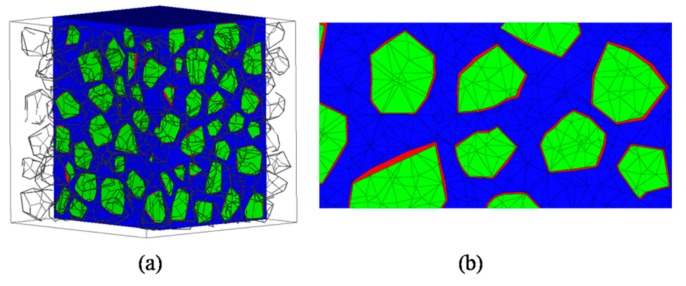
Meshing results for concrete specimen: (**a**) Two crossing planes cut view, (**b**) local enlargement cut view.

**Figure 14:**
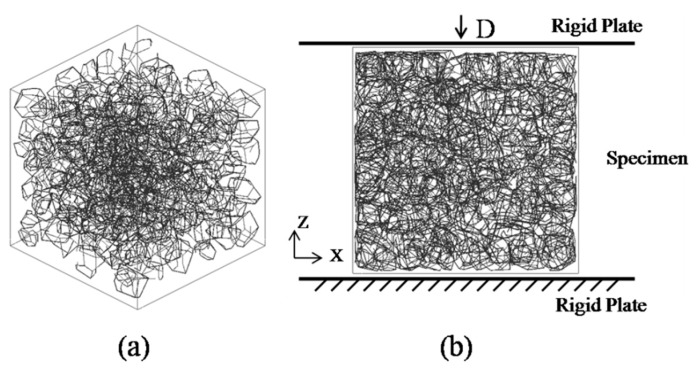
3D skeleton diagram (**a**) and schematics of sample specimen (**b**) for uniaxial quasi-static simulation.

In this work, the specimen is considered as a 25 mm side length cube with 37.38% volume fraction of aggregates. The grain size distribution is designed as in Table 2. Due to the limit of the whole specimen size, two grades are considered in this model. The smallest size of the grain is about 2.1 mm, and the largest size is about 6.3 mm, respectively. Figure 13 gives the finite element model of concrete with three phases including mortar (color of blue element), aggregate (color of green element) and ITZ (color of red element) where the thickness of the ITZ is set as 0.1 mm. Quadratic tetrahedron elements are used in the aggregate and mortar phase for ensuring the computational accuracy while quadratic wedge elements are used in the ITZ. The loading on the specimens has been applied via prescribed displacement to the rigid plate, where vertical displacements are prescribed to all nodes on the top rigid plate and six degrees of freedom are fixed on the bottom plate (Figure 14). The “D” represents the applied prescribed displacement to the rigid plate. No extra constraints have been loaded in the concrete model. In reported papers, limiting in mesh quality or magnitude, most analyses performed dynamic algorithms to solve the quasi-static problem instead of an implicit algorithm which is a better solver without the influence of iterating and loading kinematics. In this study, an implicit algorithm is employed to uniaxial compression and tension conditions using the nonlinear FE code ABAQUS/standard.

## 4. 3D Mesoscale Simulations

### 4.1. Concrete Damaged Plasticity Model

**Figure 15:**
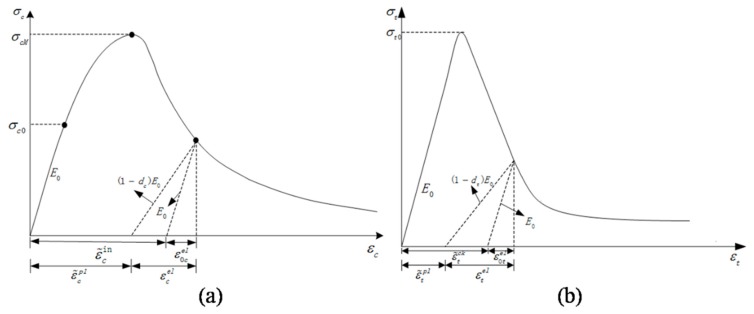
Illustration of uniaxial: (**a**) Compressive and (**b**) tensile response defined by CDP model.

The behavior of concrete is complex due to an array of morphological features as well as deformation and failure mechanisms inherent in the concrete microstructure. In recent years, many coupled plasticity–damage models have been proposed to describe the mechanical behavior of concrete [44,45,46]. The model included in the ABAQUS package (Dassault Systèmes Simulia Corp., Providence, RI, USA) called concrete damaged plasticity (CDP) has been widely used for the description of static and dynamic mechanical behaviors of concrete-like materials. The model assumes that the uniaxial compressive and tensile responses of concrete are characterized by damaged plasticity. The typical stress–stain curve is shown in Figure 15 and the stress–strain relationships under compression and tension are

(9) $$\sigma_{c}^{}=(1-d_{c}^{})E_{0}^{}(\varepsilon -{\tilde{\varepsilon }}_{c}^{pl})$$

(10) $$\sigma_{t}^{}=(1-d_{t}^{})E_{0}^{}(\varepsilon -{\tilde{\varepsilon }}_{t}^{pl})$$

where the subscripts c and t refer respectively to compression and tension; $E_{0}$ is the initial elastic modulus, ${\tilde{\varepsilon }}_{c}^{pl}$ and ${\tilde{\varepsilon }}_{t}^{pl}$ are the equivalent plastic strain; $d_{t}$ and $d_{c}$ are damage variables used to characterize degradation of the elastic modulus in the strain softening phase of the stress–strain response, and are assumed to be functions of equivalent plastic strain.

(11) $$d_{c}=d_{c}({\tilde{\varepsilon }}_{c}^{pl}),d_{t}=d_{t}({\tilde{\varepsilon }}_{t}^{pl}),0\leq d_{c},d_{t}\leq 1$$

A “pristine” stress tensor, denoted by $\overset{˘}{\sigma }$, is introduced, and refers to virtual stresses corresponding to the undamaged material state. Compressive and tensile uniaxial pristine stresses are then utilized to define the yield and failure surfaces.

(12) $$\overset{˘}{\sigma }=\left[\begin{array}{l} {\overset{˘}{\sigma }}_{c}\\ {\overset{˘}{\sigma }}_{t} \end{array}\right]$$

(13) $${\overset{˘}{\sigma }}_{c}=\frac{\sigma_{c}}{1-d_{c}}=E_{0}(\varepsilon_{c}-{\tilde{\varepsilon }}_{c}^{pl})$$

(14) $${\overset{˘}{\sigma }}_{t}=\frac{\sigma_{t}}{1-d_{t}}=E_{0}(\varepsilon_{t}-{\tilde{\varepsilon }}_{t}^{pl})$$

For actual implementation in ABAQUS, artificial plastic strain, i.e., inelastic strain ${\tilde{\varepsilon }}_{c}^{in}$ for compression (Figure 15a) and a cracking strain ${\tilde{\varepsilon }}_{t}^{ck}$ for tension (Figure 15b) are used to replace the actual plastic strain. They are defined as

(15) $${\tilde{\varepsilon }}_{c}^{in}=\varepsilon_{c}-\sigma_{c}/E_{0},{\tilde{\varepsilon }}_{t}^{ck}=\varepsilon_{t}-\sigma_{t}/E_{0}$$

The relationship between stress $\sigma_{c}$ and the inelastic strain ${\tilde{\varepsilon }}_{c}^{in}$, together with the evolution of the damage variable $d_{c}$ with ${\tilde{\varepsilon }}_{c}^{in}$, is used to define the uniaxial compressive response. Similarly, the relationships between $\sigma_{t}$ and ${\tilde{\varepsilon }}_{t}^{ck}$, and between $d_{t}$ and ${\tilde{\varepsilon }}_{t}^{ck}$, are employed to describe uniaxial tensile behavior. The actual plastic strains can be calculated from the artificial strains and damage variables, i.e.,

(16) $${\tilde{\varepsilon }}_{c}^{pl}={\tilde{\varepsilon }}_{c}^{in}-\frac{d_{c}}{1-d_{c}}\cdot \frac{\sigma_{c}}{E_{0}};{\tilde{\varepsilon }}_{t}^{pl}={\tilde{\varepsilon }}_{t}^{in}-\frac{d_{t}}{1-d_{t}}\cdot \frac{\sigma_{t}}{E_{0}}$$

When the specimen is unloaded from any point on the strain softening branch of the stress–strain curves, the elastic stiffness of the material declines.

For three-dimensional multiaxial conditions, the stress–strain relationships are govern by:

(17) $$\sigma_{}^{}=(1-d_{}^{})D_{0}^{el}:(\varepsilon -\varepsilon_{}^{pl})=D_{}^{el}:(\varepsilon -\varepsilon_{}^{pl})$$

where $D_{0}^{el}$ is the initial (undamaged) elastic stiffness of the material; $D_{0}^{el}=(1-d)D_{0}^{el}$ is the degraded elastic stiffness.

The mortar and ITZ parts are simulated using the CDP model. For normal concrete, the coarse aggregates are usually of much higher strength than the mortar parts. It is acceptable to use a linear elastic model for aggregates under quasi-static loading, but this may not reasonable for high dynamic loading such as impact and blast.

### 4.2. Statistics and Size Distribution Effect for Mesostructures

**Figure 16:**
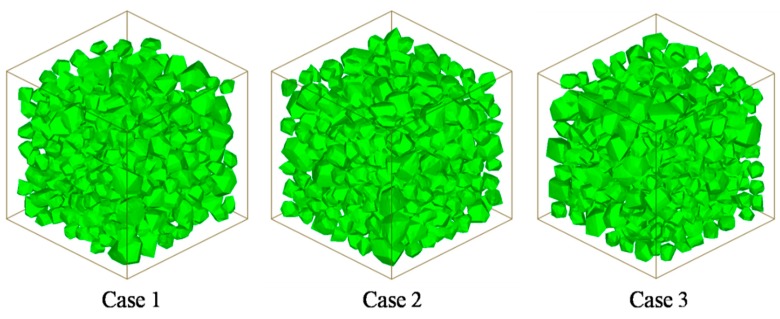
3D mesoscale model of three different size distributions.

**Figure 17:**
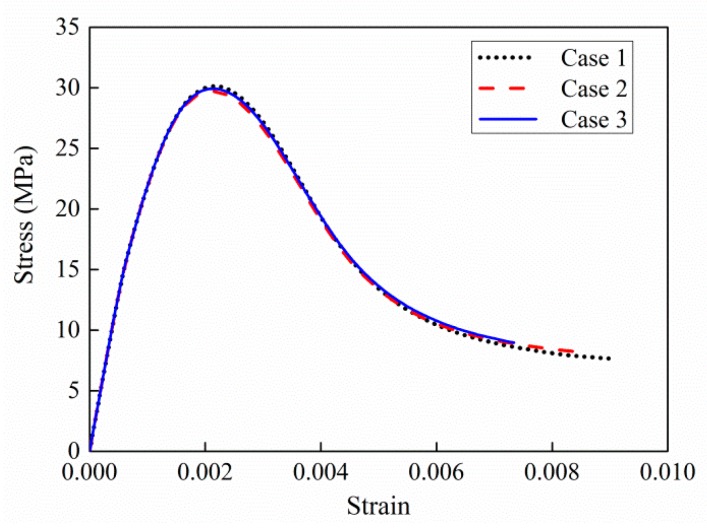
Comparison of different size distributions under uniaxial compression.

In this section, the specimen is considered as a 25 mm length cube with 30% volume fraction of aggregates. The particle size is designed as Table 3. Three mesoscale cases are plotted in Figure 16. The models have the same grain numbers and size segment but the spatial distributions are random. Figure 17 plots the simulated axial stress–strain curves under uniaxial compression. The curves are very similar in terms of curve tendency and peak stress, which illustrates that randomly spatial distributions have few influences on macroscopic strength.

### 4.3. Verification under Quasi-static Compression and Tension

The uniaxial compression tests had been performed by Van Vliet [47]. The test specimens were normal strength concrete, accordingly, the 3D mesoscale model is simulated in normal strength range. The concrete mix proportions in experiment are shown in Table 4 [47]. The grain size distribution is not absolutely identical with the experiment used as the realistic concrete includes small size gravel and sand which is really difficult to build in a numerical model and it is generally considered as mortar phase [10,20]. Nonetheless, if we consider the grades of 2.0–8.0 mm range, the proportions are similar to the simulation.

The basic mechanical parameters of the mesoscale concrete model used in current simulations are listed in Table 5. For this grade of concrete, the standard strength of mortar is around 35 MPa with Young’s modulus around 25 GPa [6,22]. As a composite material, concrete is a mixture of cement paste, aggregate with various sizes and the ITZ. Studies by other researchers showed that the ITZ has a layered structure, and it has a lower density than the bulk matrix and is more penetrable by fluids and gases [48]. Also, the ITZ appears to be the weakest region of the composite material when exposed to external loads [49]. However, it is difficult to determine the local mechanical properties in the ITZ due to the complex structure of the ITZ region and the constraints of existing measuring techniques [50]. A constant factor can be used to describe the ratio of material strength between the ITZ and mortar. Different ratio values have been employed by various researchers. The ratios employed by different works vary from 0.5 to 0.9 [33,36]. For such a thin layer of equivalent ITZ, it has been found that the strength around 70% of the mortar is appropriate [51], which is acceptable for a general range.

In the current model, 20 MPa compressive strength and 18 GPa Young’s modulus are employed for the ITZ elements. The properties of aggregates could significantly depend on the types in nature, and the Young’s modulus of aggregates is around 40–60 GPa for crushed stones [52]. Moreover, the eccentricity and the K coefficient in CDP model are set to 0.1 and 2/3, respectively. The ratio of biaxial and uniaxial compressive strength is 1.16. The viscosity coefficient is 1.0e^−5^ [53]. The density parameter is not required in the implicit solver.

**Figure 18:**
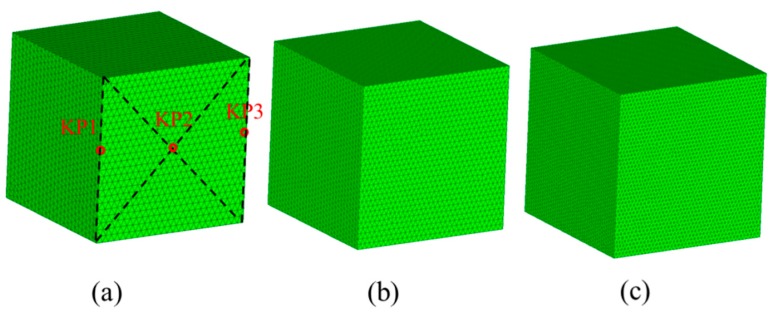
Three different mesh configurations with different mesh density: (**a**) Mesh I ($L_{e}=1\;\mathrm{mm}$), (**b**) Mesh II ($L_{e}=0.8\;\mathrm{mm}$), (**c**) Mesh III ($L_{e}=0.6\;\mathrm{mm}$).

The mesh convergence study had been performed before the uniaxial load analysis. In the test, three different average element lengths $L_{e}$ (i.e., 1 mm, 0.8 mm and 0.6 mm) were used, as shown in Figure 18. The numbers for Meshes I and II and III are 134249, 201690, and 295364 solid elements, respectively. The frictional constraint between specimen and loading plate was set as 0.1.

**Figure 19:**
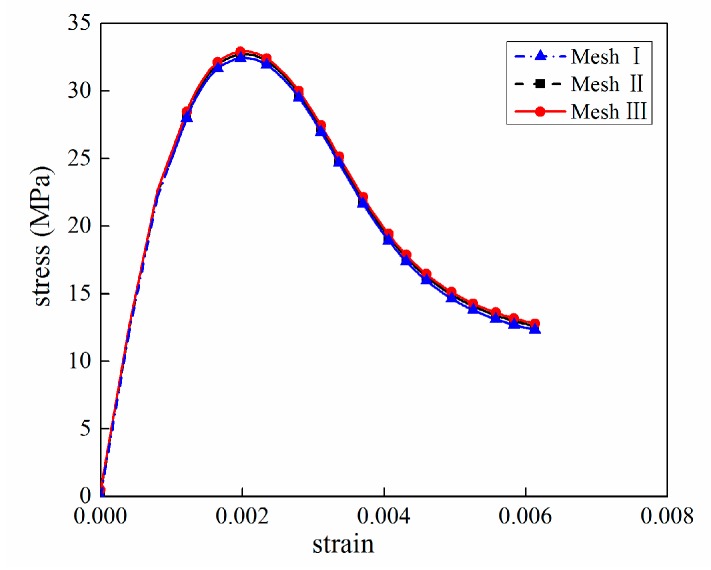
Mesh convergence results.

The simulated stress–strain curves for different element length are shown in Figure 19. Three positions named KP1, KP2, and KP3 have been marked in Figure 18a to evaluate the computational error. The stress analysis—including Mises Stress, Max Principal Stress, Mid Principal Stress, and Min Principal Stress—have been listed in Table 6, Table 7 and Table 8. The relative error is calculated by

(18) $$R=\frac{\left|S_{i}-S_{ave}\right|}{S_{ave}}$$

where $S_{i}(i=I,\mathrm{II},\mathrm{III})$ represents the Mises Stress of different mesh sizes, $S_{ave}$ is the average of $S_{i}$.

It can be observed that the relative error is less than 2%. As a result, the mesh dependence is negligible for the selected element sizes. Fine mesh would lead to large-scale nonlinear equation systems and the computational costs often become prohibitively expensive. Thus, a 1 mm element length is selected in the following analysis, where the mortar has 72114 elements and the aggregate has 36727 elements. The numbers for the ITZ phase have 25408 elements. As a reference regarding the computational cost, a mesh in 1 mm took about 6 h for compression and 10 h for tension with 18 Intel Xeon CPUs, respectively.

**Figure 20:**
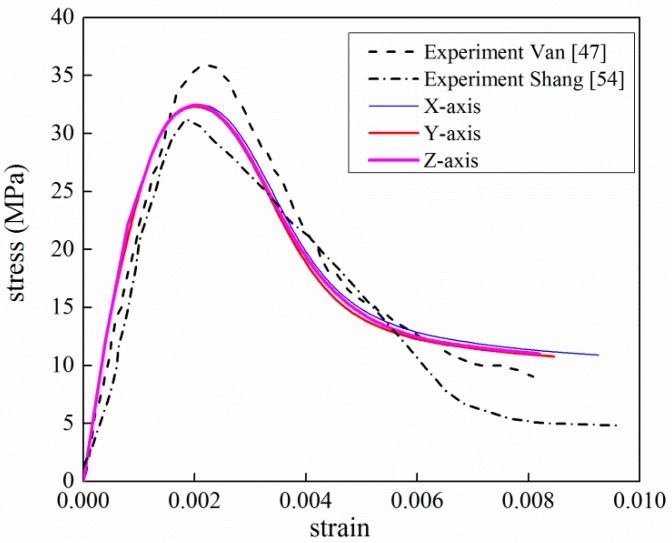
Comparison of stress–strain curves between experiment and simulation under compression (experimental data from [47,54]).

Figure 20 plots the simulated axial stress–strain curve versus experiment curve. Three loading directions of the *x*-, *y*- and *z*- axes are taken into consideration to improve the representative of numerical model. The effective stress is measured as $\frac{F_{RC}(m)}{A}$, where $F_{RC}(m)$ is the reaction force of rigid plate at the time m, A is the area of specimen surface. Respectively, effective strain is calculated as $\frac{S_{}(m)}{h}$, where $S_{}(m)$ is the displacement of rigid plate at the time m and h is the length of specimen side. As can be seen, the result from the mesoscale model shows good agreement with the experimental data in terms of peak strain and softening phase. Though the peak stress is slightly lower than the corresponding test data, the relative difference between two values is 7%, which is in an acceptable range for FE validation. Remarkably, the numerical results over-predict experimental data for strains greater than 0.006. The main reason is the damaged elements are not deleted in the loading process. In lab experiments, partial concrete splits from the test specimen. The split concrete cannot sustain any loads, which eventually results in the failure of concrete. And the stress decreases to zero. In the FE analysis, the stiffness of damaged elements decreases but cannot be zero because of the computational convergence. It means that the load capacity of finite elements is over predicted compared with the realistic concrete.

It is generally known that the compressive behavior of concrete test can be strongly influenced by the frictional constraint between the specimen and the loading platen [47]. The classical isotropic Coulomb friction model is applied in the simulation of varying frictional constraint. The model assumes that no relative motion occurs if the equivalent frictional stress $\tau_{eq}=\sqrt{\tau_{1}^{2}+\tau_{2}^{2}}$ is less than the critical stress, $\tau_{crit}$, which is proportional to the contact pressure, p, in the form

(19) $$\tau_{crit}=\mu p$$

where μ is the friction coefficient that can be defined as a function of the contact pressure.

**Figure 21:**
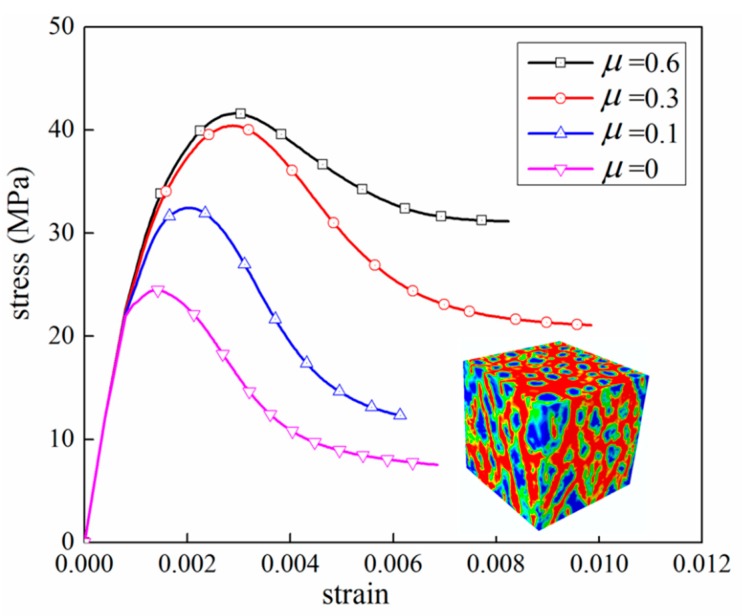
Comparison of different frictional coefficients under compression.

**Figure 22:**
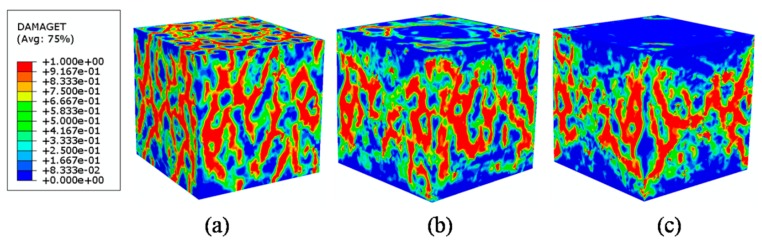
Failure patterns of different frictional coefficients μ: (**a**) μ=0.1, (**b**) μ=0.3, (**c**) μ=0.6.

**Figure 23:**
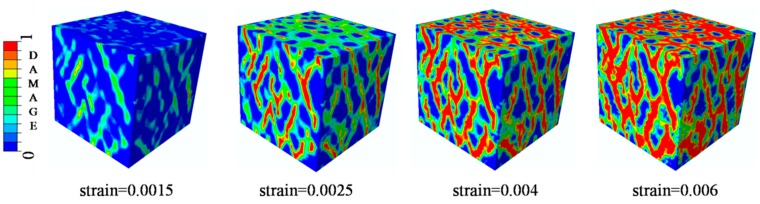
Damage evolution patterns when μ=0.

**Figure 24:**
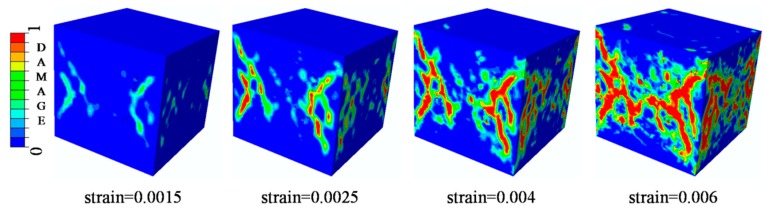
Damage evolution patterns when μ=0.6.

In the current 3D mesoscale model, it is possible to simulate the varying frictional constraint by changing frictional coefficient μ at the loading face through a surface-to-surface contact approach. For example, Guo et al. [55] studied the effect of friction between the specimen and the rigid plates. As the reference mentioned, friction coefficients from 0.1 to 0.7 is realistic in experiments, which is the range we selected in this paper. Three stress-strain curves for different μ mean 0, 0.1, 0.3, and 0.6 are plotted in Figure 21. The stress–strain curve when μ=0 shows that peak stress is obviously lower than frictional constraint. The major failure regions focus on the top surface which contacts with the rigid plate directly. It can be seen that frictional constraint has a strong influence on peak stress value and the softening phase. When the frictional coefficient increases from 0.1 to 0.3, the peak stress increases to 126%. From 0.3 to 0.6, the value of maximum stress has a little increase, but the tendency after peak stress has obvious change. Figure 22 shows the damage patterns of different μ values. In CDP models, the tensile damage factor $d_{t}^{}$ represents the degree of stiffness degradation from 0—which means no degradation, to 1—that cannot support loading completely. In other words, the output $d_{t}^{}$ from the model could be equivalent to a cracking pattern in real tests when the value of $d_{t}^{}$ is close or equal to 1. For a low frictional constraint (Figure 22a) condition, the specimen is obviously separated into a series of columns due to primary cracks almost parallel to the applied load. With μ increasing, oblique cracks rise and cracks develop in the triangular zones near the top and bottom surfaces, leading to the well-known “hour glass” failure mode (Figure 22b,c). Figure 23 and Figure 24 compare the damage evolutions with different frictional coefficients. When the μ is closer to 0, no additional lateral constrains are applied in the interaction surfaces. The lateral tensile stress, which is considered to be easier to cause the damage to concrete than compressive stress, would arise and grow to the damage threshold. The connected cracks are formed from top surface to bottom surface. With the increasing of the coefficient μ, the tangential constrains are applied on the top and bottom surfaces. Damage begins in the middle of the model. The tangential constrain limits the development of lateral tensile stress. Therefore, the damage elements cannot directly propagate parallel to the loading direction and develop to oblique cracks.

**Figure 25:**
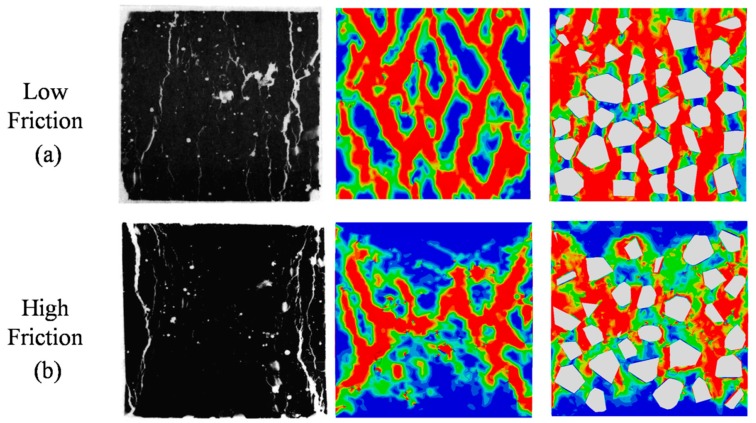
Comparison of failure patterns between experimental observations (left in each pair of graphs [56]) and numerical results (outer surface view and internal view): (**a**) Low friction condition, (**b**) high friction condition.

The comparisons of failure modes between the experiment and the simulation are plotted in Figure 25. Under the low friction condition, the vertical cracks in the experimental picture are most visible and dominate the picture of the crack pattern. The inclined cracks have opened less than the vertical cracks and are less visible. The numerical specimen is effectively separated into a series of columns by the formation of the major cracks almost parallel to the applied load, and the cracks appear to follow the weakest path along the ITZ. Under the high friction condition, confining stresses are built up in the experiment and prevent crack formation. Cracking starts from the more uniaxially stressed sides of the test specimen and results in the well-known “hour glass” failure mode. Significant confinement develops in the cone-shaped zones in the numerical results. The damage patterns appear to follow closely the weakest links formed by the ITZ around the aggregates. The crack patterns agree well with experimental observations both in low and high friction conditions, which demonstrates that the 3D mesoscale FE model is again acceptable under compressive loading.

**Figure 26:**
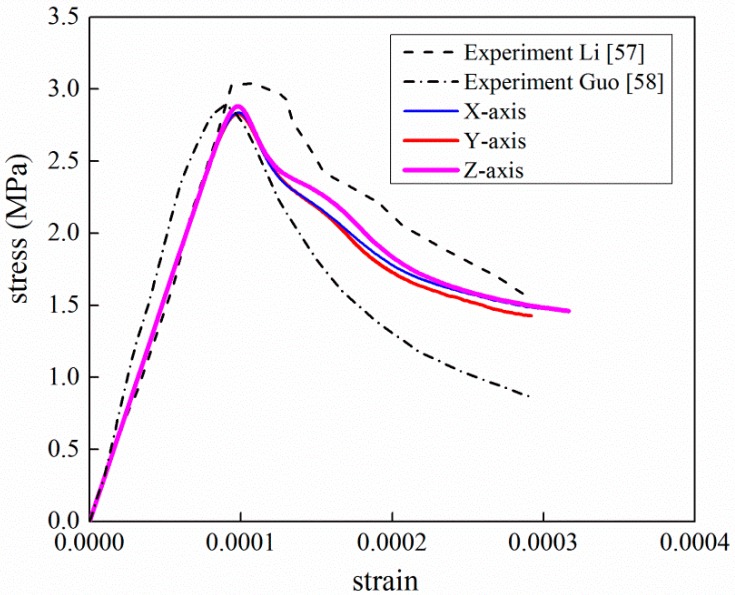
Comparison of stress–strain curves between experiment and simulation under tension (experimental data from [57,58]).

**Figure 27:**
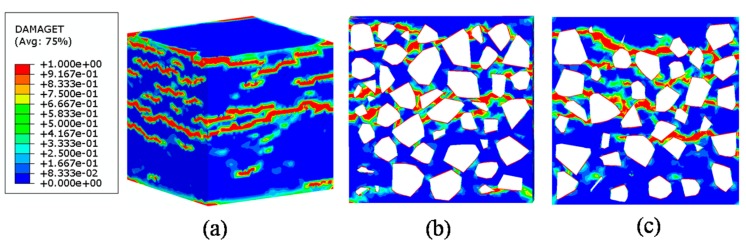
Failure patterns under tensile loading: View of 3D (**a**) and view of internal section (**b**,**c**).

The data of the tension experiment came from reference [57]. According to the reference paper, the 101 × 202 mm cylindrical tensile specimen with notches at mid-height was considered in the experiment. The top and bottom surfaces were fixed in stiff-frame by an epoxy adhesive layer. In the tensile simulation, in the present paper, a tie constraint is applied between the specimen surface and the rigid plate in order to fit the experimental boundary condition. The material parameters in the tension analysis are listed in Table 5, No lateral constraint is considered in the simulation of tension. Figure 26 depicts the comparison of test and simulation curves, and the corresponding crack mode is given in Figure 27. It can be observed that the present results agree very well with the experimental data. The crack lines are clearly perpendicular to the loading direction which is a well-known phenomenon in tension failure mode. Furthermore, the failure bands can be observed clearly through the specimen and along with the distribution change of aggregates in the section patterns. Several damage lines develop along weakest path (the ITZ phase). From the viewpoint of the internal section, the cross-cutting crack is located at the middle of specimen.

### 4.4. Analysis of ITZ Damage Evolution

**Figure 28:**
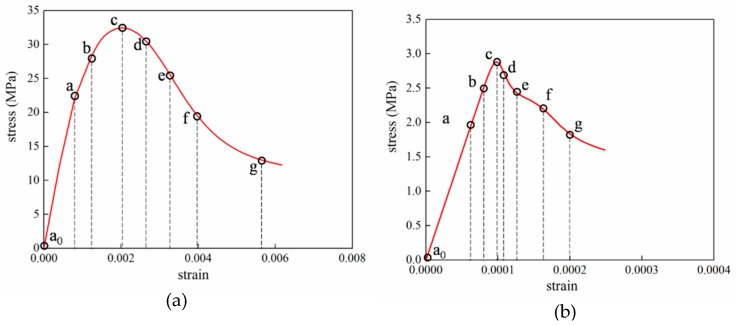
Points marked in stress–strain curves: (**a**) Compressive curve, (**b**) tensile curve.

**Figure 29:**
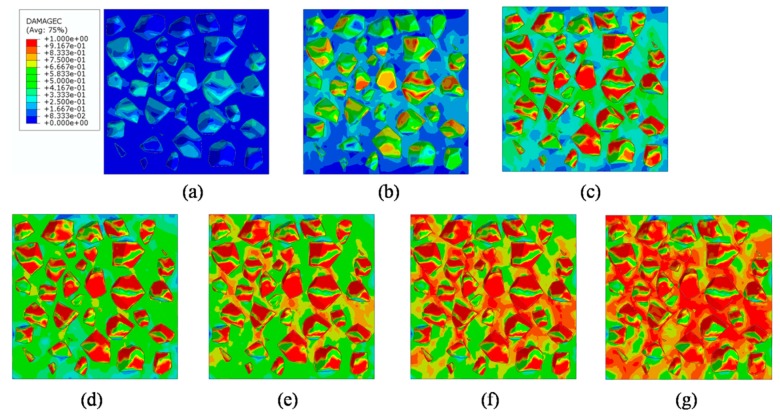
Section patterns of compressive damage initiation and evolution under compression.

**Figure 30:**
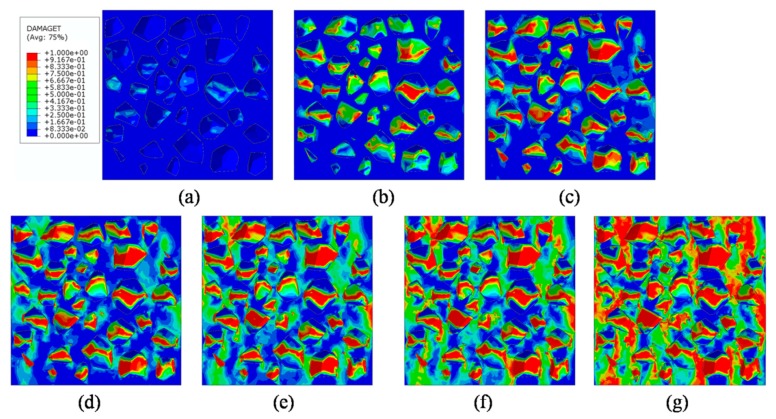
Section patterns of tensile damage initiation and evolution under compression.

**Figure 31:**
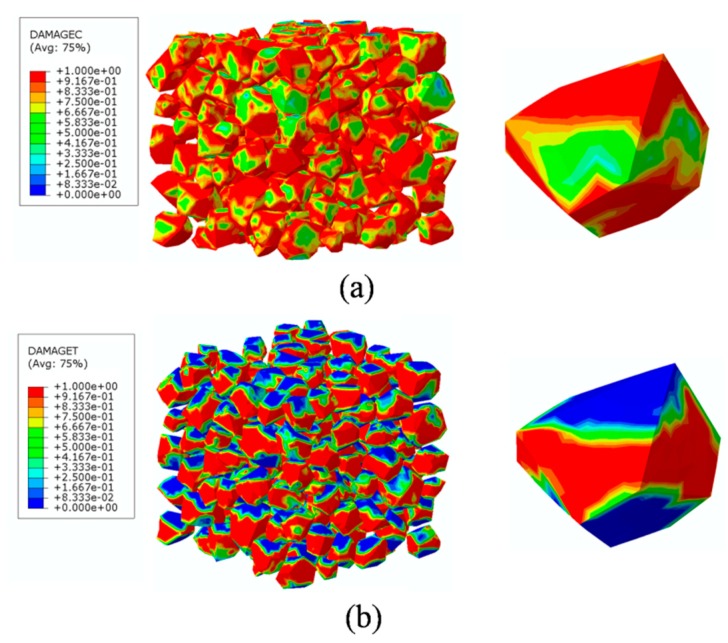
Damage diagrams of the ITZ phase at ‘‘g’’ point based on Figure 21a: (**a**) Compressive damage, (**b**) tensile damage.

**Figure 32:**
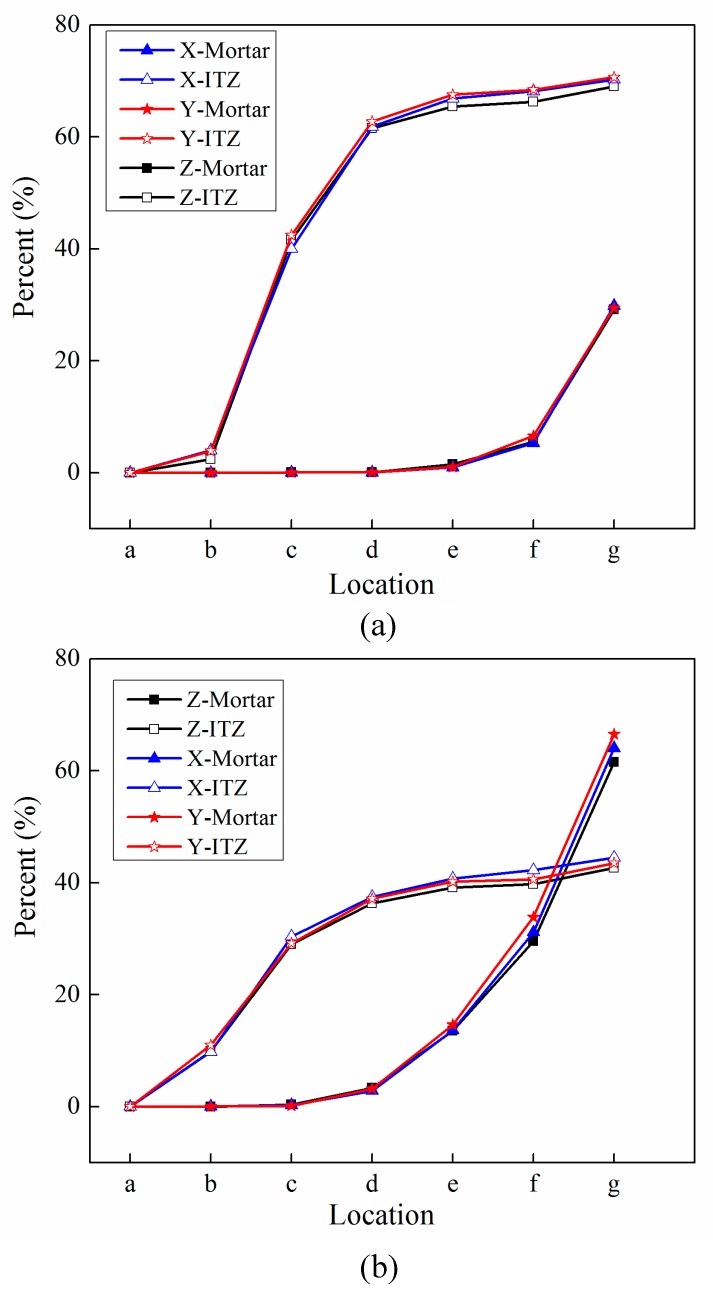
Percentage curves of damaged elements for mortar and ITZ parts under compression: (**a**) Compressive damage, (**b**) tensile damage.

**Figure 33:**
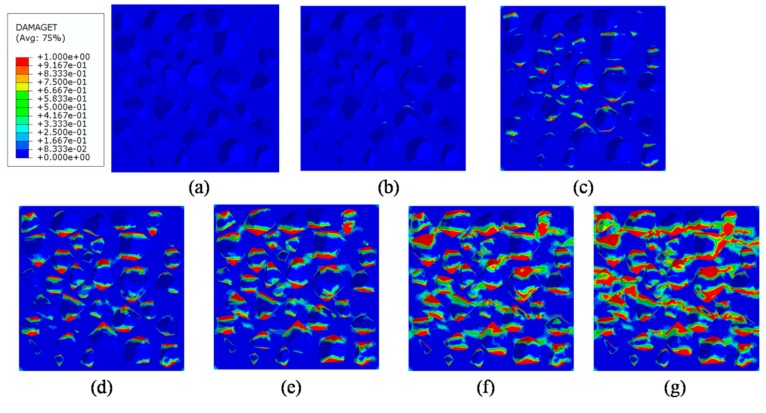
Section patterns of tensile damage initiation and evolution under tension.

**Figure 34:**
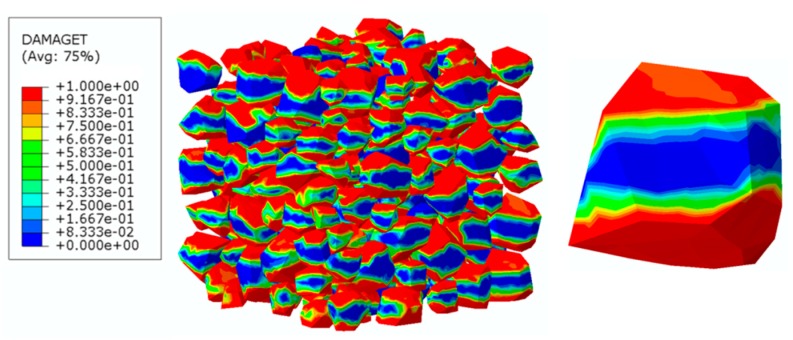
Damage diagrams of ITZ elements at ‘‘g’’ point based on Figure 21b under tension.

**Figure 35:**
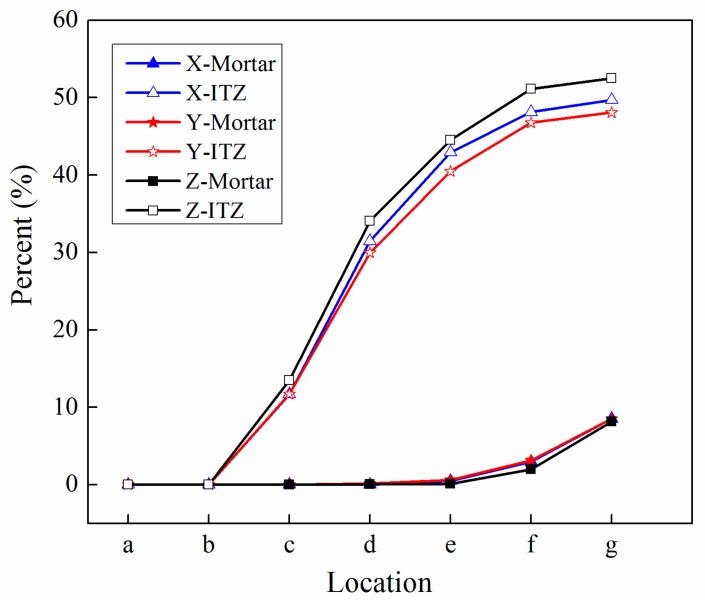
Percentage curves of damaged elements for mortar and ITZ parts under tension.

The finite element model used in this section is the same as mentioned in Figure 20. In order to investigate the damage initiation and evolution of a 3D mesoscale model under uniaxial loading, eight key points standing for different time moments during loading history are marked in the stress–strain curves both in compressive and tension condition (Figure 28). The stress–strain curves are divided three phases as elastic phase (a_0_–a), hardening phase (a–c), and softening phase (c–g). Figure 29 shows the initiation evolution (a–g) of the compressive damage factor $d_{c}^{}$ for mortar and ITZ parts. The factor $d_{c}^{}$ represents stiffness degradation from 0—with no degradation, to 1—with complete failure subjected to compressive stress. From the pictures, initial damage appears in the ITZ part firstly, and then the damage in the ITZ grows and expands to more areas. With the damage area reaching a certain range in ITZ elements, the damage bands tend to propagate towards nearby mortar parts to form a connected damage network with complicated crack bridging and branching. The process illustrates that the evolution of compressive damage starts at ITZ parts, which is considered as a weaker phase in concrete, and develops into mortar phase. The similar process is shown in Figure 30 for tensile damage factor $d_{t}^{}$. However, the damage range is really different in both the mortar and ITZ phases. The compressive and tensile damage region of the ITZ phase is plotted in Figure 31. From the specific ITZ elements around a single aggregate, compressive damage mainly distributes on the top and bottom region, while tensile damage focuses on the medium region. That means the stress state is clearly different around single aggregates under compression. The element where the computational factor $d_{c}^{}$ or $d_{t}^{}$ is greater than 0.9 is defined as an damaged element. A damaged fraction is defined as a ratio of damaged element and whole element within one material. The percentage curves of the mortar and ITZ phases under compressive condition are shown in Figure 32. Damage firstly starts in the ITZ phase and develops, while mortar phase has no damaged element until the ITZ fraction increases to a high value (almost 65% from Figure 32a). Moreover, the damaged fraction in ITZ elements is over two times that of mortar elements, which means that compressive damage mainly takes place in ITZ parts. Compared with Figure 32a, the tensile damage fraction in the mortar phase quickly increased especially on the softening range (f–g) and is higher than in the ITZ phase. In other words, tensile damage is the main cause of mortar failure. On the contrary, compressive damage is the main cause of ITZ failure from the mesoscale viewpoint. Combined with the stress–strain curve, the damaged element in the ITZ part has a quick increase around macro maximum stress. On the contrary, damaged elements in the mortar are generated until the softening phase. Figure 33 plots the initiation and evolution of the tensile damage factor $d_{t}^{}$ under an uniaxial tensile load. Initial damage starts from ITZ parts and develops to the mortar. Different from compression, the propagation range clearly concentrates on bends which is perpendicular to the loading direction. The damage diagram of whole ITZ element is plotted in Figure 34. Tensile damage focuses on the top and bottom areas around single aggregates; while in compressive condition, compressive damage occurs here. The volume percentage of tensile damaged element is shown in Figure 35. The damaged elements have a great increment in the initial softening phase. About half of ITZ elements are damaged due to the weakness of ITZ properties. The result demonstrates that tensile damage is the primary cause of ITZ failure. As quite localized cracks could lead to concrete failure, the damaged element in mortar is much less than in the ITZ under tensile loading.

## 5. Conclusions

A comprehensive procedure has been presented to establish a 3D mesoscale model for concrete. Based on Voronoi tessellation, a shrinking process is developed to separate cells into independent aggregates with a grading distribution. The in-house program called EDGE deletion is added to remove small edges, which would improve the robustness on FE meshing. The ITZ parts with different thickness can be conducted by the extending method as various factor η. Because the shrinking and extending algorithm avoids check of interaction and overlap, it is less time consuming than traditional ways in aggregates and ITZ generation.

Then the geometrical model transforms well into an FE model with higher volume content of aggregates and superior quality of mesh. As a consequence, the numerical simulation of concrete can be established using an implicit algorithm under an uniaxial load. The simulated results resemble favorably the corresponding experiment both in stress–strain curves and failure modes. For compressive simulation, different frictional constraints have great influence on the compressive progress and failure modes.

Damage evolution is investigated from the perspective of a mesoscale level under an uniaxial condition. For a compressive condition, damage initiates mostly in the ITZ and then connects to a mortar phase. Both tensile and compressive damage are well developed in different regions. For tensile conditions, tensile damage is the primary type and focuses on a localized area which is perpendicular to tensile direction. The damaged fraction is defined to generally evaluate the failure level based on element volume statistics. The results show that the mechanism of uniaxial compression and tension is very different. Tensile damage is the principal factor of mortar failure and compressive damage is the principal factor of ITZ failure under compression. On the contrary, both ITZ and mortar phase failure is dominated by tensile damage under tensile load.

## Figures and Tables

**Table 1:** Size distribution in Figure 3 [41].

Sieve Size (mm)	Total Percentage Retained (%)	Total Percentage Passing (%)
12.7	0	100
9.5	23	77
4.75	74	26
2.36	100	0

**Table 2:** Size distribution of aggregates.

Sieve Size (mm)	Total Percentage Retained (%)	Total Percentage Passing (%)
9.5	0	100
4.75	61.53	38.47
2.36	94.84	5.16

**Table 3:** Grain size of aggregates in Section 3.

Grain Diameter (mm)	Case 1	Case 2	Case 3
D_max_	6.150 mm	5.627 mm	5.869 mm
D_min_	2.334 mm	2.416 mm	2.519 mm
N1 (<2)	0	0	0
N2 (2,3.5)	260	261	265
N3 (3.5,5)	59	65	62
N4 (5,6.5)	26	15	25
N	345	341	352

**Table 4:** Concrete mix proportions.

Compositions	Concrete 1	Concrete 2
Portland Cement	330 kg/m^3^	340 kg/m^3^
Water	165 kg/m^3^	170 kg/m^3^
Gravel/Sand	1879 kg/m^3^	1828 kg/m^3^
0.0–0.25 mm	0.08	0.08
0.25–0.5 mm	0.12	0.1
0.5–1.0 mm	0.12	0.13
1.0–2.0 mm	0.1	0.16
2.0–4.0 mm	0.14	0.23
4.0–8.0 mm	0.2	0.3
8.0–16.0 mm	0.24	-

**Table 5:** Material parameters of three-phase materials.

Material	Young’s Modulus (GPa)	Poisson’s Ratio	Compressive Strength (MPa)	Tensile Strength (MPa)
Mortar	25	0.2	35	3.5
ITZ	18	0.2	20	3.0
Aggregate	43	0.23	-	-

**Table 6:** Stress statistics and relative error for different meshes on KP1.

Stress Component	Mesh I (MPa)	Mesh II (MPa)	Mesh III (MPa)
Max. Principal (Abs)	25.6776	25.6813	25.8475
Mid. Principal (Abs)	1.0868	0.4568	0.4494
Min. Principal (Abs)	0.9092	0.4009	0.1168
Mises Stress	25.6561	25.7206	26.0197
Relative Error	0.55%	0.30%	0.86%

**Table 7:** Stress statistics and relative error for different meshes on KP2.

Stress Component	Mesh I (MPa)	Mesh II (MPa)	Mesh III (MPa)
Max. Principal (Abs)	31.615	32.561	32.074
Mid. Principal (Abs)	0.512	2.353	1.491
Min. Principal (Abs)	0.013	0.628	0.909
Mises Stress	31.868	31.821	32.441
Relative Error	0.55%	0.69%	1.24%

**Table 8:** Stress statistics and relative error for different meshes on KP3.

Stress Component	Mesh I (MPa)	Mesh II (MPa)	Mesh III (MPa)
Max. Principal (Abs)	29.275	29.774	28.559
Mid. Principal (Abs)	0.578	0.495	0.185
Min. Principal (Abs)	0.247	0.435	0.141
Mises Stress	29.450	29.758	28.583
Relative Error	0.83%	0.21%	0.62%
